# Liquor Ferri Persulphatis as an Anti-periodic

**Published:** 1868-01

**Authors:** 


*Liquor Ferri Persulphatis as an Anti-periodic.*—Dr. G. H. Lenoir states (*Southern Journal Medical Science*, Nov. 1867,) that he has tried the liquor ferri persulphatis in several cases of intermittent fever, where quinia had failed, and even produced unpleasant effects.

"Immediately after the administration of the iron the chills ceased, and in but one case was there a recurrence of the malady, and in that the patient had but one chill, after which there was no symptom of a recurrence."

He gave the solution in doses of from eight to fifteen drops every four or six hours, generally preceded by a full dose of pil. cathart. comp.

